# Japanese Encephalitis Risk and Contextual Risk Factors in Southwest China: A Bayesian Hierarchical Spatial and Spatiotemporal Analysis

**DOI:** 10.3390/ijerph110404201

**Published:** 2014-04-15

**Authors:** Xing Zhao, Mingqin Cao, Hai-Huan Feng, Heng Fan, Fei Chen, Zijian Feng, Xiaosong Li, Xiao-Hua Zhou

**Affiliations:** 1 West China School of Public Health, Sichuan University, Chengdu 610041, China; E-Mails: zhaoxing731@gmail.com (x.Z.); fenghaihuan@163.com (H.-H.F.); 13211020002@fudan.edu.cn (H.F.); jacky2183@163.com (F.C.); 2 Department of Biostatistics, School of Public Health, University of Washington, Seattle, WA 98195, USA; 3 School of Public Health, Xinjiang Medical University, Urumqi 830011, China; E-Mail: cmq66@126.com; 4 Office for Disease Control and Emergency Response, Chinese Center for Disease Control and Prevention (China CDC), Beijing 102206, China; E-Mail: fengzj@chinacdc.cn; 5 HSR&D Center of Excellence, VA Puget Sound Health Care System, Seattle, WA 98101, USA

**Keywords:** Japanese encephalitis, contextual risk factors, meteorological factors, southwest China, Bayesian hierarchical model

## Abstract

It is valuable to study the spatiotemporal pattern of Japanese encephalitis (JE) and its association with the contextual risk factors in southwest China, which is the most endemic area in China. Using data from 2004 to 2009, we applied GISmapping and spatial autocorrelation analysis to analyze reported incidence data of JE in 438 counties in southwest China, finding that JE cases were not randomly distributed, and a Bayesian hierarchical spatiotemporal model identified the east part of southwest China as a high risk area. Meanwhile, the Bayesian hierarchical spatial model in 2006 demonstrated a statistically significant association between JE and the agricultural and climatic variables, including the proportion of rural population, the pig-to-human ratio, the monthly precipitation and the monthly mean minimum and maximum temperatures. Particular emphasis was placed on the time-lagged effect for climatic factors. The regression method and the Spearman correlation analysis both identified a two-month lag for the precipitation, while the regression method found a one-month lag for temperature. The results show that the high risk area in the east part of southwest China may be connected to the agricultural and climatic factors. The routine surveillance and the allocation of health resources should be given more attention in this area. Moreover, the meteorological variables might be considered as possible predictors of JE in southwest China.

## 1. Introduction

Japanese encephalitis (JE) is a mosquito-borne viral disease, which is widely distributed in South Asia, Southeast Asia, East Asia and the Pacific [1]. It is estimated that about three billion people live in countries where the JE virus is endemic [2]. JE not only causes death, but also induces permanent and psychiatric sequelae [3]. Historically, JE was serious in China. The number of JE cases has declined substantially after the long-term nationwide immunization program starting in the 1970s. However, JE still remains a significant public health issue in China, with approximately a half of the global cases annually [4], and JE virus is one of the four principal arboviruses of public health importance in China [5]. In the past few decades, the Chinese Center for Disease Control and Prevention reported that the annual numbers of death from JE ranked 2–6 among the 26 most serious national notifiable communicable diseases [6,7].

As a mosquito-borne arboviral infection, JE is caused by a flavivirus transmitted by mosquitoes of the *Culex* species. The spread of JE involves many ecological, environmental, climatic and human behavioral factors, of which two groups are significant; these are climatic factors and agricultural factors. On the one hand, climatic factors can influence the transmission of JE through their effects on mosquitoes. Temperature and precipitation have been reported to be associated with the density of mosquitoes [8]. Although suitable climatic condition could increase JE vector proliferation and longevity, the potential impact of climate change on JE remains to be investigated [2]. Particularly, like other vector-borne diseases, two common questions for JE could be whether the association with meteorological factors exists and if it does exist, what is the time-lagged pattern for the association [9]. On the other hand, two major agricultural factors affecting the JE transmission are pig rearing and the rice-irrigated areas [1,10]. Domestic pigs are the most important reservoir hosts, amplifying the transmission process [3]. The rural population has a greater chance for exposure to mosquitoes compared with the general population [11]. In addition, other socioeconomic variables may affect the transmission process, such as population immunity and housing conditions [12].

There is no specific therapy for JE other than supportive care; therefore, a better understanding of the impact from contextual factors is beneficial to prevent JE, and several studies have been conducted. In [11], it was demonstrated that at the provincial level, the risk of JE seemed to be governed by high proportions of rice-planting area, rural population and the extent of pig rearing. The authors of [13,14,15] studied the impact of the climate variability on the transmission of JE. These studies give evidence on the association between JE cases and the contextual risk factors, including agricultural and climatic factors. However, several limitations and gaps are noticeable in those studies. First, no research has been conducted to assesses the association between JE cases and the contextual risk factors in southwest China, which is the most endemic area in China [16]. The aforementioned single-site studies are in eastern China, and their results may not be valid in southwest China. Meanwhile, a better understanding of the spatiotemporal pattern of JE in southwest China would help identify areas and populations at high risk in southwest China. Second, existing studies focused on only one site to assess the association between JE and climatic variables [17]. There are two drawbacks for this approach. One is that the conclusion is hard to generalize to other sites, resulting in some inconsistent findings between the single-site studies. For example, while [14] reported that a one-month lag is the best time lag for monthly rainfall, [13,18] reported that a two-month lag is the best time lag for monthly rainfall. The other drawback is that it is not sufficient to analyze the association between meteorological factors without adjusting for agricultural variables [13,14,15]. In summary, there is no large-scale analysis (such as over 300 counties) at a small unit (such as the county level) in China to study the association between JE and the contextual factors. To the best of our knowledge, there are few large-scale studies in other countries, with the exception being in Nepal [19]. The study in Nepal analyzed the data in 2005 at the district-level to fit a spatial lag regression model with climatic, agricultural and land-cover variables.

Our aim was to assess the association between JE cases and contextual risk factors in southwest China and to identify the high risk area. This may help in understanding JE epidemiology and provide guidance for JE prevention. Specifically, we applied the Bayesian hierarchical model to explore the spatiotemporal pattern of JE in 438 counties in southwest China from 2004 to 2009. Moreover, the spatial ecological regression was conducted between JE and contextual risk factors (including agricultural and climatic variables) in 2006. This is due to the large number of cases that occurred in that year. Particular attention was given to the lag effect for climatic factors.

## 2. Materials and Methods

### 2.1. Study Region

Southwest China (21°14′ to 34°31′N, 97°35′ to 110°19′E) consists of four provinces, Sichuan, Chongqing, Yunnan and Guizhou. The area has a population of 189,977,077 (the sixth national census in 2010) and encompasses 1,137,570 square kilometers. There are 47 cities (autonomous prefectures), which can be further divided into 483 counties (county-level cities and districts).

### 2.2. Data Collection

JE monthly cases were obtained from 2004 to 2009 for the 483 counties, facilitated by the Chinese Information System for Infectious Diseases Control and Prevention (CISIDCP), which was put in place in 2004 and was more sensitive and efficient than the previous case-reporting system.

Population data for every county from 2004 to 2009 were retrieved from the National Bureau of Statistics of China. The proportion of rural population was from the statistical yearbook of these four provinces. The pig-rearing number came from the census statistics of the four provincial Bureau of Statistics at the prefecture-city level, an administrative division below a province and above a county in China's administrative structure.

County-level Geographical Information System (GIS) shapefiles of southwest China were acquired from the Chinese Institute of Geographic Sciences and Natural Resources Research.

The meteorological data contained monthly precipitation and mean temperatures, including mean minimum and maximum temperatures. They were collected from the 0.5° × 0.5° grid monthly precipitation and monthly mean temperature datasets, publicly available at the Chinese Meteorological Data Sharing Service System “http://cdc.cma.gov.cn/home.do”, which were constructed by the Chinese National Meteorological Information Center. They are meant to serve as a high resolution source of climate data interpolated from various climate sources [20,21]. We retrieved three monthly climatic variables in 2006 in the format of the 0.5° × 0.5° grid covering the entirety of China, which can be matched to each county. In general, each county only contained one grid point, and all counties took the climatic information of the grid point closest to their administrative centroids.

### 2.3. Exploratory Analysis

#### 2.3.1. GIS Mapping

All JE cases were geo-coded and matched to the county-level maps by administrative codes, and we calculated annualized incidences of JE at each county over the 6 years. For the descriptive ecological correlation analysis in 2006, the preceding geo-coding was also executed for the agricultural and climatic data. Finally, this information was visualized in the form of maps. All of the implementation above, including the spatial visualization, was accomplished by R. R is a free software programming language and a software environment for statistical computing and graphics [22].

#### 2.3.2. Spatial Autocorrelation Analysis

Global spatial autocorrelation analyses were performed for each year to discern the spatial autocorrelation of JE in southwest China after adjusting for population heterogeneity. The classic and simplest boundary-based neighborhood matrix was used. Thus, the weight is 1 if two counties share a boundary; otherwise, the weight is 0. The analysis was accomplished using R via the Assunção and Reis's global Moran's *I* statistics [23] in R package “spdep”.

#### 2.3.3. Spearman Correlation Analysis

We examined the relationship between monthly incidences of JE and monthly climatic variables over 438 counties. Spearman's correlation was used to quantify the relationship with lags from 1 to 3 months.

### 2.4. Bayesian Hierarchical Spatial and Spatiotemporal Modeling

Traditional regression methods assume that the observations are mutually independent, which is not valid, due to the spatial structure. Bayesian hierarchical spatial or spatiotemporal models [24,25] do not make such an assumption, inducing spatial correlation through random effects representing unmeasured (or perhaps unmeasurable) effects not included in the model. It can also take into account the variation of population and overdispersion [26]. These models are extensively applied to epidemiology [27].

#### 2.4.1. Spatiotemporal Model for County-Specific Relative Risks from 2004–2009

The first stage describes the observed data, *y_ijk_*, the number of cases in county *j* in month *k* of the *i*-th year, as a function of the spatiotemporal-specific relative risk of disease, *θ_ijk_*, and the expected cases, *e_ijk_*. The expected cases, *e_ijk_*, were calculated by the internal standardization method, assuming that the disease risk was constant over southwest China. A zero inflated Poisson model was employed to model the observed data, as the county × month combination would lead to excessive zeros compared to the simple Poisson model [28].

$$y_{\text{ijk}}∼\{\begin{array}{ll} \pi +(1-\pi )\times \text{Poisson}(y_{\text{ijk}}=0|e_{\text{ijk}}\theta_{\text{ijk}}) & \text{if}y_{\text{ijk}}=0,\\ \left(1-\pi )\times \text{Poisson}(y_{\text{ijk}}|e_{\text{ijk}}\theta_{\text{ijk}}\right) & \text{if}y_{\text{ijk}}=0. \end{array}$$

*π* is the probability of a zero count, and the log-relative risks log *θ_ijk_* are assumed to have the decomposition:

$$\log\theta_{\text{ijk}}=\mu +\phi_{j}+\psi_{j}+\alpha_{i}+\gamma_{i}$$

where:
*μ*:the intercept quantifying the average Poisson relative risk in the whole of southwest China*ϕ_j_*:the non-spatial random effect for the overdispersion for county *j**ψ_j_*:the spatially structured random effect for county *j*, accounting for the assumption that geographically close areas are more related than distant areas*α_i_*:the unstructured temporal effect for year *i**γ_i_*:the temporally structured effect for year *i*

More generally, we may expand this model to allow for an interaction between space and time, *δ_i_*_,_*_j_*, which would explain differences in the time trend of JE risk over the six years for different counties.

All random effects were modeled, and default minimally informative hyperpriors were set [29]. Firstly, the spatial effect was modeled as the first order intrinsic Gaussian Markov random field (IGMRF) [30]. The IGMRF accounts for spatial autocorrelation by assuming that the conditional distribution in region *i* depends on the neighboring regions, *j*. Secondly, the temporally structured effect was modeled dynamically through a time neighboring structure. Finally, the unstructured spatial and temporal effects were both specified by Gaussian models with a mean of zero. The Gamma (1, 0.0005) was chosen as the prior for the precision of the above Gaussian random effects.

#### 2.4.2. Spatial Model with Contextual Risk Factors in 2006

To examine the association between the JE relative risks and contextual risk factors, data in 2006 was used, due to the large number of cases in that year. As with [13,14], analysis was restricted to endemic months. We restricted to July, August and September, because most of the JE cases in southwest China occurred in these 3 months. The observed number of JE cases in county *j* in month *k*, *y_jk_*, was modeled as a function of the county-month-specific expected cases, *e_jk_*, and the relative risk of disease, *θ_jk_*, with *θ_jk_* allowing for the contextual factors:

$$\log\theta_{jk}=\mu +\phi_{j}+\psi_{j}+\beta_{1}\times x_{j(k-l_{1}),1}+\beta_{2}\times x_{j(k-l_{1}),2}+\beta_{3}\times x_{j,3}+\beta_{4}\times x_{j,4}$$

where *μ*, *ϕ_j_* and *ψ_j_* are the same as the spatiotemporal model; the others denote the contextual variables as follows:
*x_j_*_(_*_k_*_−_*_l_*_1),1_:the temperature of the (*k* − *l*_1_) month for county *j**x_j_*_(_*_k_*_−_*_l_*_2),2_:the precipitation of the (*k* − *l*_2_) month for county *j**x_j_*_,3_:the proportion of rural population for county *j**x_j_*_,4_:the pig-to-human ratio for county *j*

Here, *l*_1_ and *l*_2_ represent the lagged effects for the temperature and the precipitation, respectively, and they varied from 1 to 3 months. Moreover, similar to [13], the monthly mean minimum and maximum temperatures were modeled separately, as the two temperatures are highly correlated.

The random effects were exactly the same as the spatiotemporal model. The default highly dispersed Gaussian distribution with the mean equal to 0 and the variance equal to 1,000 was specified for all the fixed effects, *β*_1_ to *β*_4_.

Two groups of models were fitted using a forward stepwise procedure with an increasing complexity. The first group only included climatic variables to choose the best time lags, while the second model further included the proportion of rural population and the pig-to-human ratio, in order to investigate the association. A Berkson-type measurement error model was used for the pig-to-human ratio. Berkson-type error occurs in biological or epidemiological studies, where averages of exposures in areas are assigned to individuals living or working close-by [31]. Roughly speaking, the method was used to deal with the multiple resolution of the exposure variables. The pig-to-human ratio was observed at a coarser level [32,33], the prefectural level, as opposed to the county level. The prefecture-city level is an administrative division below a province and above a county in China's administrative structure. All covariates were standardized to eliminate the scale effect and to improve the accuracy of parameter estimates.

#### 2.4.3. Computation and Model Choice

All models were computed using integrated nested Laplace approximations (INLA), a method for approximate Bayesian inference within latent Gaussian models. INLA outperforms traditional Markov chain Monte Carlo (MCMC) in terms of computational time, while keeping very precise results [34]. Analyses can be performed using R-INLA, available at “http://www.r-inla.org/”. Competing models were compared and selected using deviance information criterion (DIC), and a smaller DIC indicates a better trade-off between the model fit and complexity [35].

## 3. Results

### 3.1. Descriptive Results

A total of 17,007 JE cases were reported in southwest China from 2004 to 2009. There were 10,489 male and 6,518 female cases. Children under five years old accounted for 48%, 5- to 15-year-old school children for 45.8% and those > 15 years old for 6.2%.

**Figure 1:**
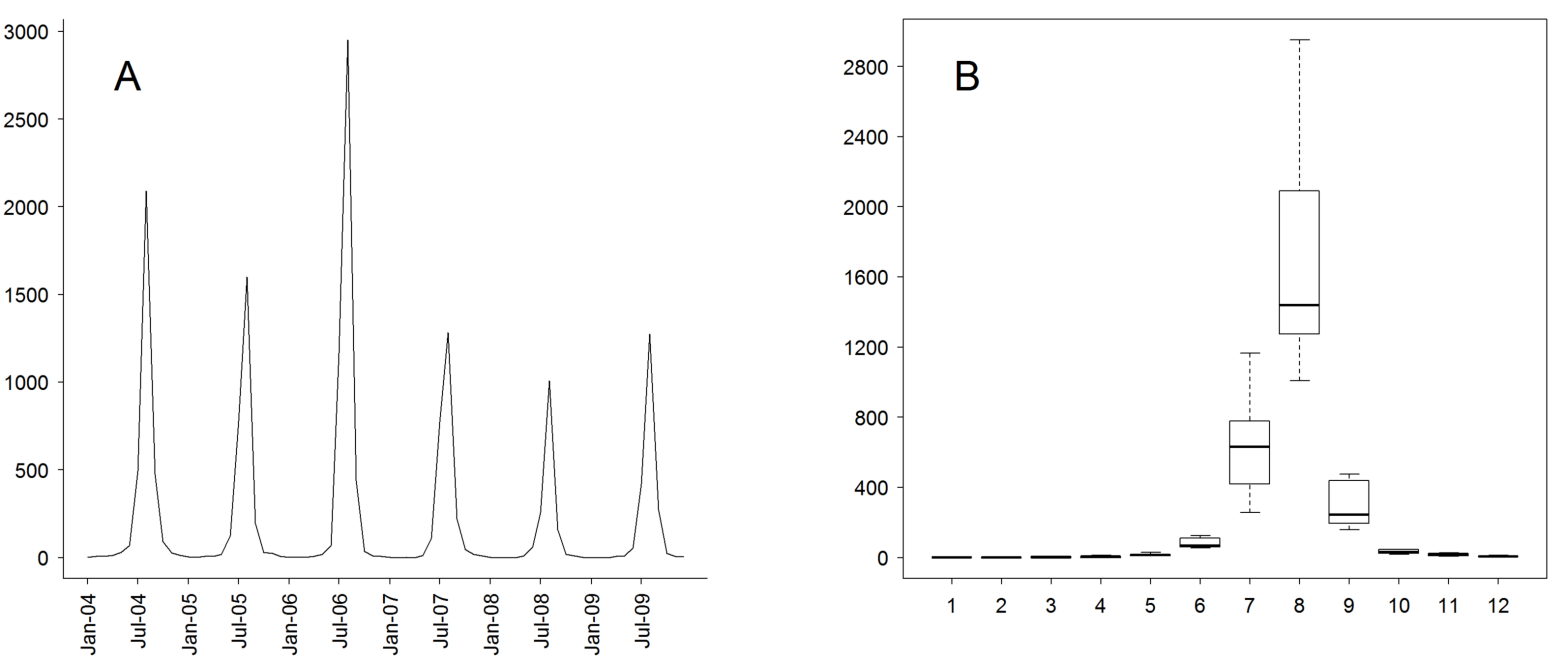
Temporal distribution patterns of Japanese encephalitis (JE) cases in southwest China from 2004 to 2009. (**A**) The figure shows the epidemic curve of monthly JE cases; (**B**) the seasonal epidemic patterns of the JE distribution. The bottom and top of the box indicates the lower quartile (*P*_25_) and the upper quartile (*P*_75_), respectively; the line in the middle of the box represents the median value; whiskers represents 1.5 times the height of the box.

Figure 1 shows a time series of JE cases, a significant seasonal peak with 83.3% of cases occurring in July–August, and 93.8% in July–September. Furthermore, the annual incidence peaked in 2006.

**Figure 2:**
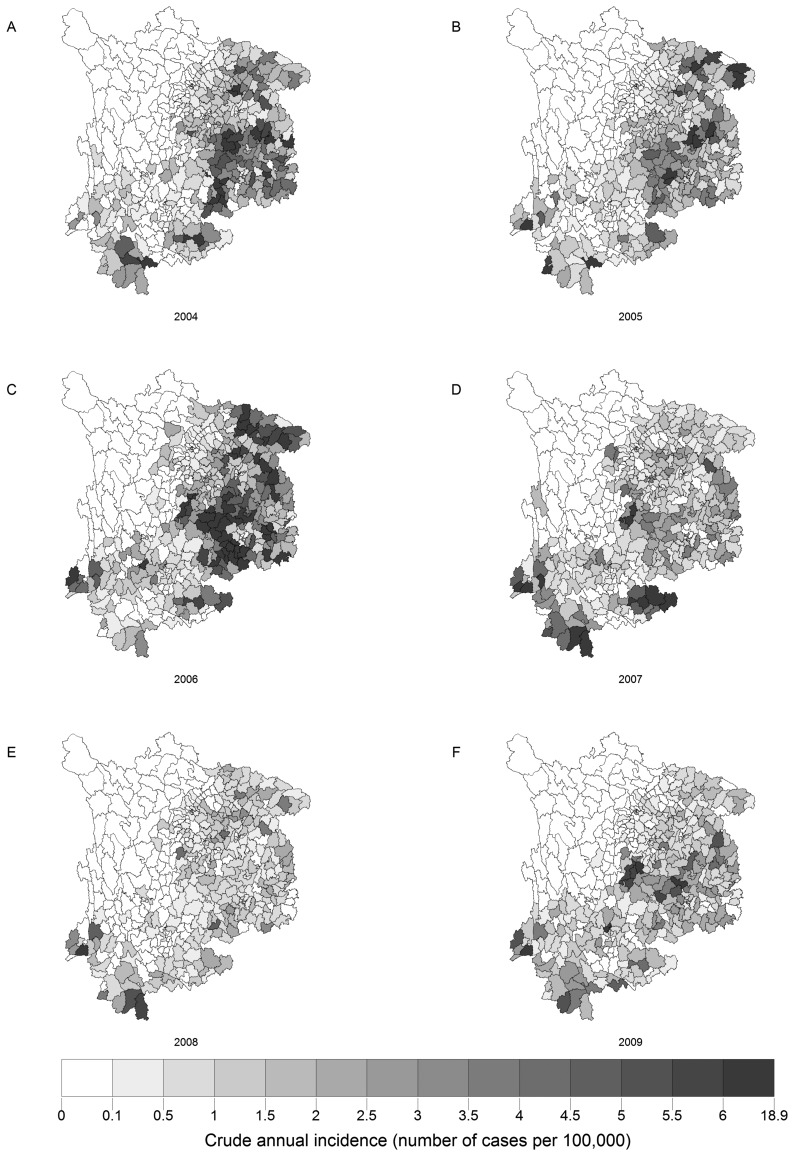
Annualized incidence of JE maps in southwest China from 2004 to 2009. The figure shows the crude annual incidence (number of cases per 100,000) in the following years: (**A**) 2004; (**B**) 2005; (**C**) 2006; (**D**) 2007; (**E**) 2008; (**F**) 2009.

Figure 2 demonstrates annualized incidences of JE maps from 2004 to 2009, while Table 1 presents spatial autocorrelation analyses of JE for each year. They show that Moran's *I* was significant for each year, implying that the distribution of JE was spatially autocorrelated in southwest China. Moreover, Moran's *I* of the first three years is significantly higher than those of the subsequent three years, implying the autocorrelation became smaller later. This is also evident from Figure 2.

**Table 1:** Spatial autocorrelation analyses for the annualized incidence of JE in southwest China from 2004 to 2009.

**Year**	**Moran's** ***I***	***p*-value**	**Pattern**
2004	0.5196	0.001	Clustered
2005	0.5014	0.001	Clustered
2006	0.5146	0.001	Clustered
2007	0.3555	0.001	Clustered
2008	0.2945	0.001	Clustered
2009	0.3874	0.001	Clustered

**Figure 3:**
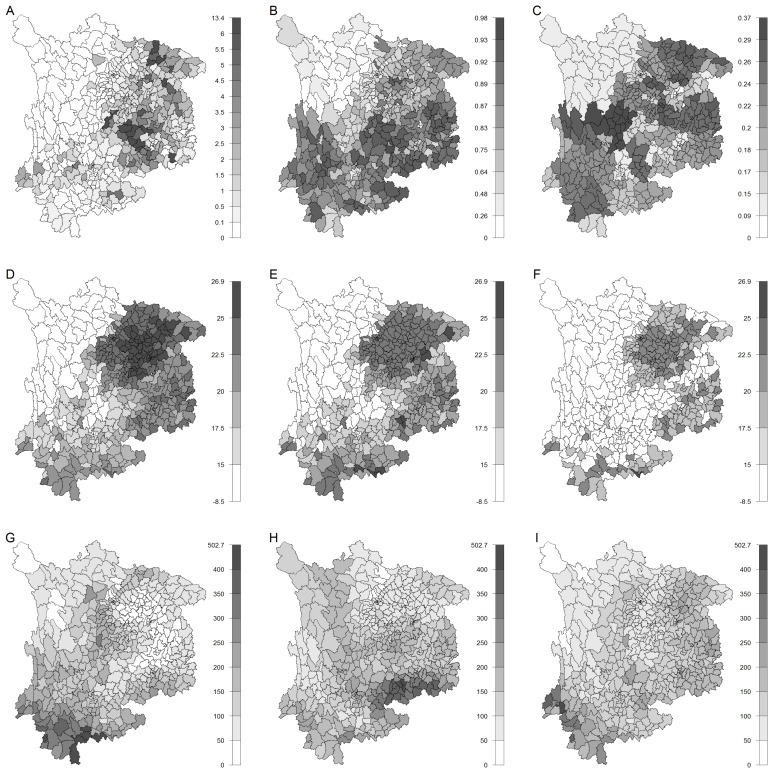
County-level information in southwest China in 2006. The figure shows the following information in 2006 at the county level: (**A**) the crude monthly incidence in August (number of cases per 100,000); (**B**) the proportion of rural population; (**C**) the pig density (the number of pigs per 1,00 people); (**D**) the monthly mean minimum temperature in July; (**E**) the monthly mean minimum temperature in June; (**F**) the monthly mean minimum temperature in May; (**G**) the precipitation in July; (**H**) the precipitation in June; and (**I**) the precipitation in May.

Figure 3 presents the following county-level information in 2006: the crude incidence rates of JE in August, the proportion of the rural population, the pig-to-human ratio, the monthly mean minimum temperature and precipitation, with month lags varying from one to three to August. These graphs show several features. Firstly, the JE cases generally concentrated in the east area. Secondly, there is some degree of similarity between the spatial pattern of JE cases and those of the mean minimum temperature, the proportion of the rural population and the pig-to-human ratio. Thirdly, the geographical pattern for the precipitation did not show a clear correlation with JE cases. Finally, the spatial patterns of the precipitation varied from June to August, while the geographical pattern of the mean minimum temperature was preserved with continuing change in the magnitude. Here, to preserve space, we just reported the monthly mean minimum temperature. Similar results could be found for the monthly mean maximum temperature.

Table 2 shows the Spearman correlation coefficients between monthly incidences (from July to September) and the three climatic variables with different time lags in 2006. The highest positive correlation constantly occurred with a two-month lag for the precipitation, whereas the correlation for the two temperatures does not show a clear pattern.

### 3.2. Bayesian Hierarchical Spatial and Spatiotemporal Modeling

#### 3.2.1. Results of Spatiotemporal Model for County-Specific Relative Risks from 2004–2009

**Figure 4:**
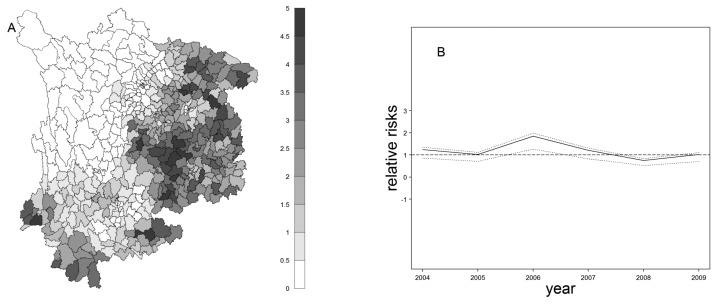
Spatiotemporal trend in southwest China from 2004 to 2009. (**A**) The posterior mean of county-specific excessive relative risks and the sum of spatially and non-spatial random effects; (**B**) the excessive temporal trend, posterior mean of year-specific excessive relative risks and the sum of temporally structured and unstructured effects.

Two different spatiotemporal models were fitted, with and without a space-time interaction. The model with the interaction had a greater fit (DIC = 26,281.63) than the model without interaction (DIC = 28,529.48). Figure 4A presents the posterior mean of county-specific excessive relative risks, the sum of spatially structured random and non-spatial random effects. This reflects the excessive spatial trend of disease risks for the 438 counties after allowing for sparse counts and correlation effects, and it is evident that high risk areas are located in the eastern and that some are far southern areas. Figure 4B shows the excessive temporal trend posterior mean of year-specific excessive relative risks and the sum of temporally structured and unstructured effects. Overall, JE presented a declining temporal trend in southwest China, with a peak in 2006.

**Table 2:** Spearman correlation coefficients between monthly incidences and climatic variables with different time lags, from July to September in 2006.

**Month of incidence**	**Precipitation**			**Monthly mean minimum temperature**			**Monthly mean maximum temperature**		
		
	**1-month lag**	**2-month lag**	**3-month lag**	**1-month lag**	**2-month lag**	**3-month lag**	**1-month lag**	**2-month lag**	**3-month lag**
July	0.233 **	0.432 **	0.423 **	0.331 **	0.335 **	0.332 **	0.258 **	0.268 **	0.131 **
August	0.139 **	0.237 **	0.187**	0.258 **	0.259 **	0.245 **	0.221**	0.229 **	0.101 **
September	0.063	0.087 *	−0.038	−0.067	−0.074	−0.077	−0.051	−0.055	−0.063

** The difference between the correlation coefficient and zero is statistically significant, *p* < 0.05;

* the difference between the correlation coefficient and zero is statistically significant, *p* < 0.1.

#### 3.2.2. Spatial Model with Contextual Risk Factors in 2006

First, DICs were used to select the best time lags for meteorological variables. Table 3 reports the DICs of models with different month lags for rainfall and temperature. The first column “precipitation” shows the DICs of models with rainfall as the only covariate, and the best fit model is the two-month lag precipitation, with DIC being 8614.96. The result is consistent with the Spearman's correlation analysis. Together with the two-month lag precipitation, different month lags for the monthly mean minimum temperature were modeled. The result is reported in the second column, and the one-month lag presents the best fit, with DIC equal to 8503.39. The monthly mean maximum temperature shows a similar result with the best fit with DIC equal to 8508.47. The minimum temperature shows a slightly better fit than the maximum temperature by the comparison of DICs.

Table 4 presents estimated parameters of models with spatial random effects and all contextual factors, including agricultural variables and the selected time-lagged climatic variables. Monthly mean minimum and maximum temperatures were modeled separately. The two models show a pronounced increase in model fit over the models just with climatic covariates, with the DIC decreasing by about 34% from a comparison of Table 3 and Table 4. Besides, as before, the model with monthly mean minimum temperature has a greater fit (DIC = 5470.98) compared to the model with monthly mean maximum temperature (DIC = 5702.9). These contextual variables all show a statistically significant positive association with JE, as their 95% credible intervals are over zero. Although the monthly mean minimum and maximum temperatures are not directly comparable as they are in different models, the former presents a greater regression coefficient over that of the latter, 1.097 (C.I.= 0.979, 1.222) *versus* 0.483 (C.I. = 0.408, 0.559). Since all contextual variables are standardized, this may suggest that the variation of the minimum temperature has a greater association with JE cases compared to the maximum temperature. This is consistent with the result of DICs. Furthermore, in both models, the regression coefficients of temperature are greater than the precipitation. For example, in the model with the minimum temperature, the coefficient for the monthly mean minimum temperature is 1.097, while the coefficient for the precipitation is 0.261. Since all independent variables were standardized, this may imply that the variation of temperature is more important for JE variation than precipitation. Moreover, examining Figure 3 shall lead to the same conclusion, as temperature shows a remarkably greater geographic similarity with JE than the precipitation. As expected, coefficients for the proportion of rural population and the pig-to-human ratio are both statistically positively significant.

**Table 3:** Deviance information criteria (DICs) of models with different month lags for the precipitation and temperature in 2006.

**Time lag**	**Precipitation**	**Monthly mean minimum temperature +2-month lag precipitation**	**Monthly mean maximum temperature +2-month lag precipitation**
1-month	8,707.21	8,503.39	8,508.47
2-month	8,614.96	8,614.06	8,554.03
3-month	8,618.33	8,582.91	8,616.4

**Table 4:** Parameter estimates of models with agriculture related variables and the selected time-lagged climatic variables, in addition to the spatial random effects.

**Variable**	**Model with monthly mean minimum temperature**				**Model with monthly mean maximum temperature**			
	
	**mean**	**SD**	**95%**	**Credible interval**	**mean**	**SD**	**95%**	**Credible interval**
temperature	1.097	0.06	0.979	1.223	0.483	0.039	0.408	0.559
precipitation	0.261	0.024	0.215	0.307	0.235	0.023	0.19	0.28
rural	0.35	0.091	0.172	0.529	0.311	0.084	0.15	0.48
pig	0.242	0.087	0.067	0.41	0.333	0.081	0.177	0.485
intercept	−1.017	0.101	−1.22	−0.819	−0.797	0.103	−1	−0.566
		
DIC	5470.98				5702.9			

## 4. Discussion

Japanese encephalitis still remains one of the major national arboviral diseases in China [5] and is mainly located in southwest China [36], which is our focus. Unlike existing studies in a single site, our study used data from 438 counties in southwest China. Besides, our analysis was relatively comprehensive by including meteorological and agricultural variables.

There are two main findings in the current work. First, spatiotemporal analysis has identified the east part of southwest China as a high risk area and that area may be connected to agricultural and climatic factors. Based on the ecological regression analysis, the high risk area is associated with the high pig-to-human ratio, a high proportion of rural population and weather change. An unpublished thesis [37] had analyzed the association between agricultural factors and JE in the southwest of China at the prefecture-city level, an administrative division below a province and above a county in China's administrative structure, but found no statistically significant factors. Including a comprehensive set of variables and the county-level analysis are the unique characteristics of this study. The model-based approach confirms the association between JE and contextual risk factors. Since southwest China is a less-developed area compared with the other parts in China, it has limited funds for JE control. These identified high risk areas should get the priority in allocating health resources to promote the existing surveillance, infrastructure redevelopment and in-house workforce training. Two strategies may be implemented for pig-rearing, pig immunization and the separation of pig rearing from human settlements. Besides, monitoring the infection rate in pigs may supply information for possible outbreaks.

Second, the time-lagged association between JE incidences and temperature and precipitation are quantified with an adjustment for other agricultural variables. It is important to study the impact of climate on JE transmission, because global warming might change the patterns of temperature and precipitation, which may influence the development of mosquitoes and the virus.

Minimum and maximum temperatures are found to be positively associated with JE incidences, which is biologically plausible. Temperature can affect three aspects of JE transmission: the survival and reproduction rates of mosquitoes, the biting rate of mosquitoes and the development of JE virus within mosquitoes. Thus, higher temperatures, within limits, can lead to the quicker development of larvae, shorter times between blood meals and faster incubation times for viral infections within mosquitoes. As a result, higher temperatures cause mosquito populations to reach a higher density faster and to be maintained for a longer period, thereby increasing the chance for viral transmission [38].

The minimum temperature results in a better model fit and a greater regression coefficient compared to the maximum temperature, implying that the minimum temperature presents a greater effect on JE transmission compared with the maximum temperature. Minimum temperatures might play a more important role for larvae survival in cold conditions. Similar results were reported for other vector-borne diseases [39,40,41].

Precipitation is found to be positively associated with JE incidences. Precipitation would bring a high relative humidity, which may increase the propagation and development of mosquitoes. High rainfall can also provide water support for the development of mosquitoes at the larval and pupal stages.

The one-month lag for temperature is consistent with [14,18], and the two-month lag for precipitation is consistent with [13,18]. There are several periods to be considered for the lagged effect, such as the time for mosquitoes to develop, the development period of JE virus within the mosquito and the incubation period of the virus within the human body. The lag times are distinct between temperature and rainfall, with a shorter lag for temperature. This might result from the difference regarding the biological mechanisms. The temperature can contribute to the transmission of JE through its effect on the development of mosquitoes and the viruses within them, whereas rainfall may just affect the mosquitoes. Hence, temperature may present a faster and stronger effect. The stronger effect can be observed from Figure 3 and the regression coefficient in Table 4, in which the variable temperature factors demonstrate a larger regression coefficient than the precipitation. The interaction between temperature and rainfall was not included in the model for two reasons. First, it is not unreasonable to assume there is no such an interaction, which is the usual assumption from most existing studies for vector-borne diseases [17,42]. Second, the preliminary exploratory analysis did not show a significant interaction.

The relationship between climate and JE may be highly dependent on local environmental factors, and it is not always possible to extrapolate to a broader scale [15]. Therefore, existing results from other sites may not be appropriate for southwest China. Due to the severe consequences of JE, surveillance for the early warning of epidemics based on meteorological factors in combination with agricultural variables may be of paramount importance in southwest China. Our findings give a better understanding for the JE epidemiology in this area. When designing a JE early warning system in southwest China, health departments should consider the lag patterns for meteorological factors, as well as the strongest impact from minimum temperatures.

The limitations of this study should be acknowledged. Firstly, some factors were not included in the ecological regression, such as the population immunity (including vaccination), the area of the irrigated land, the socioeconomic status and the control measures for mosquitoes. Unfortunately, those data were unavailable over the 438 counties in 2006. The hierarchical Bayesian spatial method was used as an effort to better estimate the parameters for the observed variables by allowing for unmeasured covariates [43]. However, the potential bias cannot be eliminated. Secondly, only the data in 2006 was used to investigate the association, as it is very difficult in practice to collect all variables over the 438 counties for the six years.

## 5. Conclusion

Climate change, especially global warming, has already brought and will continue to bring about challenges for infectious disease control [44]. In our work, a county-level analysis identified the east part of southwest China as the high risk area, which may be connected to agricultural and climatic factors, and therefore, the routine surveillance and the allocation of health resources should be given more attention in this area. Pig immunization and the separation of pig rearing from human settlements may also assist in limiting the transmission. Moreover, the monthly mean minimum and maximum temperatures and monthly rainfall showed lagged correlations with JE. Temperature demonstrated a greater correlation than rainfall, and the minimum temperature presented a greater model fit than the maximum temperature. The climatic factors might be considered as possible predictors of JE in southwest China.
